# Mammary Jacket: A Paper Read before the Montgomery Medical, Society, Tennessee, on the 3d of May, 1851

**Published:** 1858-12

**Authors:** D. C. Higbee


*Mammary Jacket: A Paper read before the Montgomery Medical, Society, Tennessee, on the 3d of May,* 1851. BY D. C. HIGBEE, M. D.

Mammary Abscess is a disease which, for many reasons, we are all taught to dread; but I would as gladly treat it as almost any other abscess of like extent, wherever located. Indeed, I believe that it is as easily controlled as any other.

In the limbs, the neck, and in the parietes of the abdomen and chest, we are troubled with impeded muscular action, and extension of the sac, from the ease with which the muscles are dissected up; and per chance a skilful surgeon only can open it with safety; besides, it may be impossible to collapse it by mechanical means, as in psoas abscess, abscesses on the anterior regions of the neck, those of the abdominal parietes, etc. Not so with mammary abscess. Any veterinarian may lance it, any negress may bandage and cure it.

I do not pretend to offer any new principle: it is only a better application of one well known. Compression by means of the common roller, adhesive strips, etc., requiros the skill of experience to adjust it properly; nor should the trouble, anxiety, and loss of time and comfort, be lost sight of. Should you use the common roller, or the adhesive plaster, you are debarred from the use of nearly all other local remedies, which a peculiar case might require.

The *first* and the great indication in the treatment of abscesses, is to collapse them; and to do this they should be freely opened, if possible, that the pus may all find exit. I cannot suppose that we disagree here.

In May, 1843, I was called to attend Mrs. L—— (in Lexington Ky.) for this disease. She was small, delicate and scrofulous; ætat. 23; second child, and second attack. My treatment accorded with that of "our fathers." Two months passed and my patient greatly worse, notwithstanding I availed myself of experienced consultation.

At this time I stumbled on the treatment by MM. Trousseau and contour. They recommended the treatment by strips of adhesive plaster, the complicated application of which was nearly equal to Dessault's dressing for fractured clavicle. However, my bedridden patient was thus relieved in some four weeks more, to me the only redemption was a long bill, whilst her consolation must have been that I did not kill her.

In such cases I generally used the resinous adhesive plasters, but I found it necessary to re-dress the mammæ very frequently, owing to the perspiration, pus, milk, etc., loosening them. Many of you, I doubt not, have been equally annoyed; if so, you know too well the pain inflicted on our patients.

The old method of treating this disease I will not detail, feeling assured you will all join me in saying that it is very unsatisfactory.

Having the principles of the bandage as taught me by that prince of the roller, (Dr. Dudley,) whose merits have been so justly won, and whose honors so justly crowned the praises of Transylvania, and having the hints of MM. Trousseou and contour, I conceived the following simple method of treating this disease in all its stages:

Take a strip of strong cotton, woollen, or other goods, from ten to fourteen inches wide, and sufficiently long to allow the end to overlap each other by several inches after encircling the chest. To this are sewed two broad and short shoulder-straps, having places hollowed out of the broad piece so that undue pressure will not be made in the axillæ. Neither splints nor starch need be used to keep the jacket from rolling upward; the configuration of the chest will always keep it smooth: just the reverse will be obtained in the abdominal bandage, in parturition. This jacket is to be put on like a vest, (but next the shin,) except when the shoulder-straps are secured in front by a strong pin, which is generally preferable, that we may change the position of the mammæ with ease, or that the patient may be saved the trouble of undressing when a clean one is desired.

Now suppose the abscess already well opened. If you wish to apply a poultice, let it be smoothly spread, circular, and *generally thicker at the base of the gland*, or otherwise, as the peculiar shape of the breast may require. When they are made thickest at their edges, the pressure on the gland will be most equalized, for the circumscribed force, acting laterally on its base, will prevent its becoming more flattened than natural, which I consider necessary to avoid when the gland is involved. Let us never lose sight of the anatomy of this organ.

When I am not using the poultice, I surround the breast with wool or cotton to fill the same indication. But what I should greatly prefer in many cases would be a thin caoutchouc bottle of globular shape or a very large bladder, so that when partially filled with some liquid or air, it might be semi-collapsed, and laid over the mammæ, and banage over it, which would insure a uniform pressure on all parts. If there be but one breast diseased, cut a sharp oval hole lengthwise with the bandage, or jacket, through which the healthy breast may protrude, having the aperture just large enough, so that there will not be undue pressure on the gland; thus the patient may give suck without inconvenience.

Now let the lower edges or the jacket be drawn firmly around the body and secured by pound pins. Then place your hand under the mammæ and poultice, and elevate them rather above the point they would occupy if the patient were in a recumbent posture. If the cavity be very great, it may be necessary to draw the breast laterally, to place the sinus or lips in a position favorable for resolution, or for the complete escape of purulent matter, or for both. Continue the pinning of the jacket from *below upward*, until its ends are well secured.

My rule for tightening this jacket is this: let it be as tight as the patient can comfortably bear it. If the inflammation be very acute, and the tumefaction great, a slight pressure only may be tolerated at first, but she will soon be able to bear it tighter from time to time, as the irritability, congestion, and inflammation subside.

In nearly every case this jacket should be adjusted when the patient is recumbent, for the gravity of the ponderous breast will thus be conveniently overcome. This being done, cut an opening, if necessary, through which the nipple may protrude, having already secured one through the poultice, that the milk may be drawn with a breast-pipe or nipple-glass, by a nurse, or even the child, should the lacteal ducts not be diseased. The dressing should always be renewed speedily, and that, too, *when the patient is recumbent.*

By this mode of treatment I have never failed in performing a cure, in all ordinary cases, in from five to fourteen days, and the worst case I have ever seen has never required more than three weeks.

I will give only one bad case. I could give many, but I consider it unnecessery to occupy your time.

A negro girl, Bet, aged 10, good constitution, second child, and second attack of mammary abscess. When I was called in she had been laboring under this disease about six weeks. She was much emaciated, slept but little, loss of appetite, digestive apparatus very torpid, etc— The breast was of enormous bulk; three sinuses perforated the fascia and gland, each one large and deep enough to admit the whole of my fore-finger; indeed, the gland appeared to be wholly disorganized. I applied the jacket, and gave simple medicines to correct the digestive organs.

No opiates were given; the mechanical pressure lessens the pain more effectually than any anodyne can. Her improvement began with the application of the jacket, and in three weeks her breast was well. I then directed her to wear the jacket one month longer, and during the time to loosen it more and more, until finally it would be no support.

Here, then, is an ignorant negress *nursing herself*, adjusting her jacket, with no assistance whatever.

But decidedly the greatest utility of this jacket is obtained, as I conceive, in using it as a

PROPHYLACTIC.

Whenever my patient is threatened with mammary abscess, I apply it as above, and I have never failed in attaining my object—I mean when I could apply it during that stage which Sir Astly Cooper denominates the "adhesive stage." To be more explicit:

1st. Sir Astley is of opinion that the "draught" is the principal cause of this disease.

2d. Churchill says that sore nipples may produce it.

3d. Burns says, "Some have their breasts prodigiously distended when the milk first comes, and the hardness extends even to the axillæ. If in these cases the nipple be flat, or the milk do not run freely, the fascia, particularly in some habits, rapidly inflames. Others are more prone to have the dense substance in which the acini and ducts are imbedded, or the acini themselves, inflamed."

4th. It is not unfrequently the case that congestion and irritation will retard the period of lactation, and if abscess has previously existed, there will be great danger of its recurrence if neglected ; and especially is this the case in many constitutions.

5th. Some patients have a great dread of the lancet, and will not submit to its use ; then, if the abscess be small, or very deep-seated, or if there be doubt that suppuration has set up, it might be very objectionable to lance it.

Under *all* the above circumstances I have used the jacket with entire satisfaction, both to myself and patients, MM. Trousseau and Contour think that if pressure be continued too long when the suppuration is very active, the pus may be disseminated over a large surface. Now I am at a loss to conceive how this can readily take place when the lateral pressure at the base of the gland is equal to or greater than the vertical preasure on its apex. We must remember that well-adjusted pressure will retard purulent secretion when very copious, and generally it will even cause an absorption of what already exists.

But let us as at all times be governed by facts. That there may be cases where the jacket would be an evil, I will not dispute, yet I have never seen one. Emetics may fail to produce emesis in some people, whilst in others hyperemesis may be induced. Is this an argument against their use at all? Certainly not. Then, as I would use the bandage in fracture or in aneurism, to fill certain indications, so would I use it in inflammation of the breasts, whether attended or not by abscess. But shall we exclude all other remedies for the jacket? By no means. Let us use in conjunction with it our nauseants, purgatives, opiates, tonics, alteratives, etc., and our cooling and astringent lotions, whenever indicated.

Sir Astley speaks of "the induration which sometimes continues for a long time after the abscess has healed," and for this he recommends frictions with camphorated mercurial ointment, iodine ointment, soap liniment, with tincture of iodine, etc. Use these, if you please, in conjunction with the jacket, but the latter I would consider *sufficient of itself.* I ask, what is the modus operandi of friction in such indurations? Nearly the same as mechanical pressure, they differing only in degree. But, as I before remarked, this induration is never a sequela after the proper use of the jacket; but should it occur, I would expect to remove it by the means above detailed, and without any other agent.

Permit me now to make a brief *synopsis*

1st. This jacket is the best known means for using poultices and various other remedies.

2d. It is so *simple* in its use that the patient can treat herself after being taught the rules to be observed and the objects to be attained. Indeed, so fond do they generally become of this remedy, that they lay it aside with reluctance, even after they are well.

3d. When properly adjusted, the pain will be greatly mitigated; the patient can sleep sweetly, roll from side to side in bed, nurse, work, and can assume almost any attitude without inconvenience.

4th. It retains the diseased parts in the most favorable position possible, for rapid resolution.

5th. It checks purulent secretion, and generally causes an absorpton, partial or complete, of what already exists.

6th. By early resolution you avoid all the evils of a protracted case, which, in many constitutions, if they do not bring about the death of the mother or child, are nearly as intolerable.

7th. Suppose half the gland to be already destroyed: collapse the sac, and heal as speedily as possible: it is better to be crippled than martyred.

8th. Perhaps many of the tumors, and other diseases of this organ might be cured by compress; and why not? Have we not found the common roller already removing very many diseases which were formerly the prey of the scalpel, or which resisted all the materia medica? Need I mention aneurisms, ulcers, mobid growths etc.?

9th. And lastly, a certain amount of blood flowing to the breasts is necessary for a speedy and healthy secretion of milk. More or less than a given amount of arterial circulation will lessen the amount of milk secreted in a direct ratio. If there be a stasis of blood or of milk, or if the mother has lost her child, or from bad health or other causes she is compelled to dry up her breasts, or should she have more secreted than the child requires, or her health should justify, in *all such cases* use the jacket properly, and you will very seldom find use for your saline purgatives, low diet, etc., etc.

On many occasions where mothers gave too little milk for their babes, I have quite succeeded in producing an abundance by advising them to be a little more *unfashionable*, to make the bodies of their dresses larger, and to remove the whalebones, so that the mammæ will not be continually pressed. Such women (*à la mode*) you will find to give most milk during their hours of dishabille.—*Nashville Medical Record.*

